# Lifetime prevalence and clinical correlates of nonsuicidal self-injury in youth inpatients with eating disorders: a retrospective chart review

**DOI:** 10.1186/s13034-022-00446-1

**Published:** 2022-02-28

**Authors:** Sabine Arnold, Antonia Wiese, Sarah Zaid, Christoph U. Correll, Charlotte Jaite

**Affiliations:** 1 Department of Child and Adolescent Psychiatry, Psychosomatic Medicine and Psychotherapy, Charité – Universitaetsmedizin Berlin, corporate member of Freie Universitaet Berlin, Humboldt Universitaet zu Berlin, and Berlin Institute of Health, Berlin, Germany; 2 Department of Psychiatry, The Zucker Hillside Hospital, Northwell Health, Glen Oaks, NY USA; 3 Department of Psychiatry and Molecular Medicine, Zucker School of Medicine at Hofstra/Northwell, Hempstead, NY USA

**Keywords:** Nonsuicidal self-injury, Eating disorders, Bulimia nervosa, Anorexia nervosa, Youth

## Abstract

**Background:**

Youths with eating disorders (EDs) engaging in nonsuicidal self-injury (NSSI) are at higher suicide risk because EDs and NSSI are associated with suicidality. However, epidemiologic data on NSSI lacks in the vulnerable group of youth ED inpatients.

**Methods:**

This retrospective chart review included patients up to 18 years of age with an ICD-10 diagnosis of anorexia nervosa, restricting type (AN-R), anorexia nervosa, binge-purge type (AN-BP), and bulimia nervosa (BN), treated at the child and adolescent inpatient department of the University Hospital in Berlin, Germany, between 1990 and 2015. Across and within ED subgroups, lifetime NSSI prevalence, methods of self-harm, and clinical correlates were evaluated. Independent correlations of demographic and clinical factors with NSSI were identified via multivariable regression models.

**Results:**

Of 382 inpatients (median = 15.6 (range = 9–18) years, females = 97.1%), 21.5% reported lifetime NSSI, consisting of cutting = 86.6%, scratching = 12.2%, and hitting = 8.5%. NSSI was more frequent in BN (47.6%) and AN-BP (39.3%) than AN-R (8.3%) (Φ = 0.43). Across ED subgroups, NSSI was associated with a higher prevalence of psychiatric comorbidities (AN-R: Φ = 0.55; AN-BP: Φ = 0.69; BN: Φ = 0.78), suicidal ideation (AN-R: Φ = 0.30; AN-BP: Φ = 0.38; BN: Φ = 0.29), and psychiatric medication use (AN-R: Φ = 0.23; AN-BP: Φ = 0.64; BN: Φ = 0.60). In multivariable regression analyses, NSSI was independently associated with a higher prevalence of psychiatric comorbidities (AN-R: OR = 2.93 [1.42, 6.04]; AN-BP: OR = 2.67 [1.13, 6.31]; BN: OR = 3.75 [1.71, 8.23]). Additionally, independent correlates with NSSI in AN-R included a higher prevalence of suicidal ideation (OR = 0.21 [0.72, 0.64]) and less weekly weight gain (OR = 0.03 [0.02, 0.43]), while in BN, NSSI was correlated with longer inpatient treatment duration (OR = 1.01 [1.00, 1.02]).

**Conclusions:**

There is a high lifetime prevalence of NSSI among youth with AN and BN requiring inpatient treatment, especially those with binge-purge behaviors. Treatment programs must be tailored to address psychiatric comorbidities and suicidality to improve patient care and suicide prevention.

*Trial registration:* This study was not considered a clinical trial but a retrospective chart review based on routinely assessed clinical parameters. The study includes data from human participants, however: (1) no intervention and no prospective assignment to interventions were performed, and (2) no evaluation of an intervention on participants was accomplished.

**Supplementary Information:**

The online version contains supplementary material available at 10.1186/s13034-022-00446-1.

## Background

Eating disorders (EDs) and nonsuicidal self-injury (NSSI) are associated with increased suicide risk [1–3], creating a serious public health concern, especially when EDs and NSSI co-occur. NSSI captures the deliberate destruction of one's skin without a conscious intention of dying for purposes socially not approved [4–6]. Alarmingly, even if engaged in NSSI without suicidal intent, NSSI strongly correlates with suicidality [7–11]. Underlying psychological functions of NSSI may serve affect-regulation and disruption of dissociation and suicidal ideation [2, 12, 13]. Accordingly, body piercing or tattooing is not conceptualized as NSSI, while self-cutting one's skin to relieve high emotional stress is [5]. Alarmingly, the prevalence of NSSI appears to be increasing in adolescents these days [11].

EDs include anorexia nervosa (AN) and bulimia nervosa (BN) [15]. Specifically, AN comprises a restricting (AN-R) and a binge-purge (AN-BP) subtype and characterizes a self-induced, clinically significant underweight status accompanied by weight phobia, body image distortion, and hormonal deviations. BN is characterized by repeated binge eating and behavior counteracting potential weight gain, for example, self-induced purging [15]. Severely ill patients with AN and BN treated in specialized inpatient settings showed enhanced mortality [16], with suicide as one of the leading causes of death [3, 17].

EDs and NSSI share several risk factors and functions [18]. Both are self-destructive, have the onset and peak incidence during youth, are more prevalent in females, and share underlying functions of affect regulation and increased mortality risk by suicide [2, 19–22]. From an etiological perspective, the attachment pattern appears to be crucial. Specifically, in adolescent ED inpatients, disorganized attachment status was associated with NSSI [23]. This association can be explained by an unresolved attachment pattern, often resulting from adverse childhood experiences, that causes emotion regulation difficulties when confronted with interpersonal attachment distress, with NSSI serving to resolve emerging aversive emotions and tension [23]. Apart from this, the increased suicide risk in EDs co-occurring with NSSI can be explained by the interpersonal theory of suicide [24, 25]. According to this theory, repeated pain exposure, for example, through cutting or starvation, reduces the primary reaction, such as fear, and amplifies the opposite valance response, such as relief, which may facilitate suicide. Taken together, minors with AN and BN engaging in NSSI and treated in an inpatient setting may be a particularly vulnerable population. More differentiated epidemiological data on NSSI in this exposed group is crucial to improve patient care and suicide prevention.

## Lifetime prevalence of nonsuicidal self-injury in anorexia nervosa and bulimia nervosa

As an essential epidemiological measure, lifetime prevalence indicates the proportion of individuals affected by a disease at any point in their lives. Prevalence estimates reveal the disease burden and are crucial to administer adequate health care [26, 27], including accessible treatment by qualified staff. Since NSSI is stigmatizing and epidemiological knowledge is scarce, obtaining prevalence data is critical, especially for vulnerable population subgroups.

A recent meta-analysis summarizing research on NSSI in EDs quantified lifetime NSSI prevalence as 27% across different age groups and treatment settings ([20]; 29 studies). Specifically, in adult in- and outpatients with AN-R, AN-BP, and BN, approximately half engaged in NSSI [28, 29]. Concerning minors suffering from AN and BN, only four studies focused on lifetime NSSI. In particular, in a child and adolescent sample studied by Wiederman et al. [30], NSSI was found in 29.3% of patients with BN (n = 58) and 14.6% with AN (n = 59) (mean age = 15.4 ± 1.4 years). Likewise, a history of NSSI was reported by 38.1% of BN (n = 23) and 14.7% of AN (n = 34) (mean age = 16.9 ± 1.6 years) in an analysis of Ruuska et al. [31]. Another study conducted among adolescents and young adults revealed a lifetime NSSI prevalence of 43.2% in AN-BP (n = 47), 32.4% in AN-R (n = 223), and 20.8% in BN (n = 169) (mean age = 15.4 ± 1.9 years) [32]. These three studies examined adolescent and young adult outpatients. However, only one recent study included partially hospitalized patients, reporting lifetime NSSI in 87.5% of BN (n = 8) and 30.0% of AN (n = 100) (mean age = 14.3 ± 1.7 years) [33].

Overall, the broad range of lifetime NSSI varying between 15 and 88% could result from different treatment settings, populations, and measurements based on diverse NSSI conceptualizations [34–36]. Higher NSSI prevalence in binge-purge EDs [29, 37] could be explained by affect regulation for the increased impulsivity and negative affect in BN and AN-BP vs. AN-R [38, 39]. Taken together, only four studies were identified focusing on a history of NSSI in adolescents and adults with EDs. However, their sample size was limited, especially for BN, the prevalence was often not reported for ED subgroups, and the AN subtypes were mainly merged. Moreover, to the best of our knowledge, no study analyzed lifetime NSSI in youth inpatients, comparing AN-R, AN-BP, and BN.

Detailed knowledge of the prevailing NSSI method in specific samples, considering psychiatric disorder, age group, and treatment setting, is crucial for tailoring screening efforts and care. Regarding the prevalence of different NSSI types across adolescent and young adult community samples of ED outpatients, previous studies almost consistently described cutting as the most common method, followed by scratching, hitting, and burning [13, 28, 32, 40–42]. However, it is unclear which NSSI methods are predominantly used by youth requiring inpatient care for their ED, comparing AN-R, AN-BP, and BN.

## Clinical correlates of nonsuicidal self-injury in anorexia nervosa and bulimia nervosa

Knowing which clinical phenomena co-occur with a major psychiatric condition is crucial for identifying individuals at risk, offering prevention at an early stage, and tailoring treatment programs. It is essential to understand the clinical correlates of NSSI as a high-risk behavior that is related to fatal outcomes.

Two reviews [2, 19] and several recent individual studies including youths and adults from the community and non-ED-specific psychiatric in- and outpatient settings identified the following clinical correlates of NSSI: female sex [10], family psychopathology [43], history of childhood physical abuse [43], emotional abuse [43], and sexual abuse [2, 19, 43], as well as a higher prevalence of comorbid psychiatric disorders [11], namely substance abuse [2, 19], mood disorder [19], depressive symptomatology [2], anxiety [2, 19], obsessive-compulsiveness [19], borderline personality disorder [2, 19], suicidal ideation [10, 11, 19], and suicide attempts [2, 10, 11, 19].

Moreover, specific risk factors for NSSI were identified for ED populations. According to a systematic review ([3]; 66 studies) and recent individual studies of youths and adults with EDs, NSSI was associated with female sex [32], older age [32], higher body weight [32], severe ED psychopathology [3, 41], a longer duration of illness [32], a longer treatment duration [13], a history of childhood physical abuse [3, 44, 45], emotional abuse [3, 40, 45], and sexual abuse [3, 44, 45]. Further, NSSI was associated with more psychopathology [13, 31], substance abuse [3, 32], affective disorders [32], major depression [3, 13, 31, 44], anxiety disorders [31, 45, 46], obsessive-compulsiveness [3, 29, 41], post-traumatic stress disorder [19], personality psychopathology [37], including borderline, histrionic [45], and not otherwise specified personality disorders [13]. NSSI correlated with more suicidal ideation [45] and suicide attempts [3], as well as antidepressant [47, 48] and antipsychotic [48] medication prescription.

An earlier ED onset and lower weight gain in AN were associated with a poorer disease outcome, which is associated with NSSI [21, 49]. However, the association between ED onset, weight change during treatment, and NSSI prevalence is not clear yet. Moreover, in previous studies, NSSI was associated with less efficiency [46], more academic distress [50], and lower academic performance [51]. However, the association between NSSI and IQ in youth ED inpatients has remained unclear. While adjustment problems were associated with an increased risk of NSSI [52], the prevalence of NSSI in ED patients with comorbid adjustment disorder is not known yet. Moreover, according to the Werther effect, learning about suicide can trigger own suicidal tendencies, and NSSI is used as an anti-suicide skill [2]. However, the association between the prevalence of attempted or committed suicides in the environment of youth ED inpatients and NSSI remains unclear.

In general, specific psychopharmacological therapy of NSSI is not recommended by the German guidelines [53]. In acute cases, practitioners occasionally resort to anxiolytic medication, such as benzodiazepine, for sedation when a patient suffers from severe internal tension and a strong urge to engage in NSSI [54, 55]. Still, the association between NSSI prevalence and the frequency of anxiolytic medication prescriptions in youth ED patients requires further study.

## Study aims

To date, very few studies have analyzed the lifetime prevalence of NSSI in youths with EDs. Prevalence estimates varied considerably, probably due to small sample sizes, treatment settings, and different NSSI measurements. Moreover, only one outpatient study compared prevalence across the AN subtype. Further, we are not aware of research on the course of NSSI in patients with EDs.

To close these gaps, this study addressed the following research questions in youth inpatients across and within ED subtypes: (1) What is the lifetime prevalence of NSSI? (2) What is the predominant NSSI method? (3) Which variables are independently associated with NSSI? Based on the available literature, we hypothesized that (1) lifetime NSSI is more prevalent in BN and AN-BP than in AN-R, (2) cutting is the primary NSSI method, (3) higher ED-disease severity, higher prevalence of female gender, psychiatric comorbidities, psychiatric medication use, and suicidality are significantly associated with NSSI. Due to a lack of prior research on this, we were unable to formulate specific hypotheses regarding demographic and psychopathological variables as correlates of NSSI frequency in ED subtypes. Beyond, we explored time trends of lifetime NSSI prevalence across ED subgroups within the 25-year time frame of the retrospective study.

## Methods

### Study design and participants

**Fig. 1:**
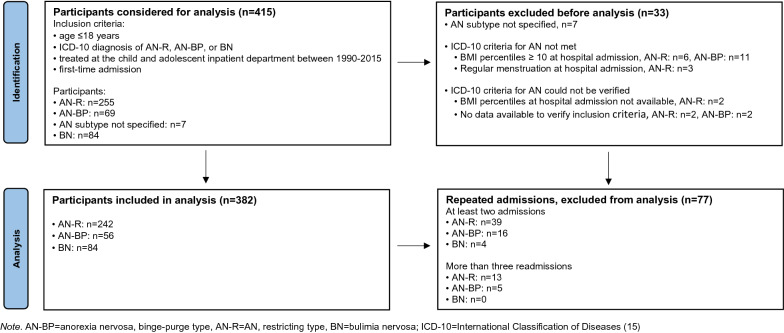
Study flow chart of the retrospective cohort study design

This retrospective chart review examined consecutively admitted patients treated at the Charité University Hospital child and adolescent psychiatry inpatient department in Berlin, Germany. All records of patients up to the age of 18 years (collectively called "youth") with an ED diagnosis of AN-R, AN-BP, and BN (ICD-10; [15]), treated between 1990 and 2015 (the period when the departmental computerized data collection template utilized for data capture was in place) were evaluated. In the case of multiple hospital admissions, only data of the first stay were taken into account (please see Fig. 1). Patients with atypical ED diagnoses were excluded from this study. Patients received an ED-specific, multimodal treatment, including nutritional counseling, body therapy, group, and individual psychodynamic or cognitive-behavioral psychotherapy.

**Fig. 2:**
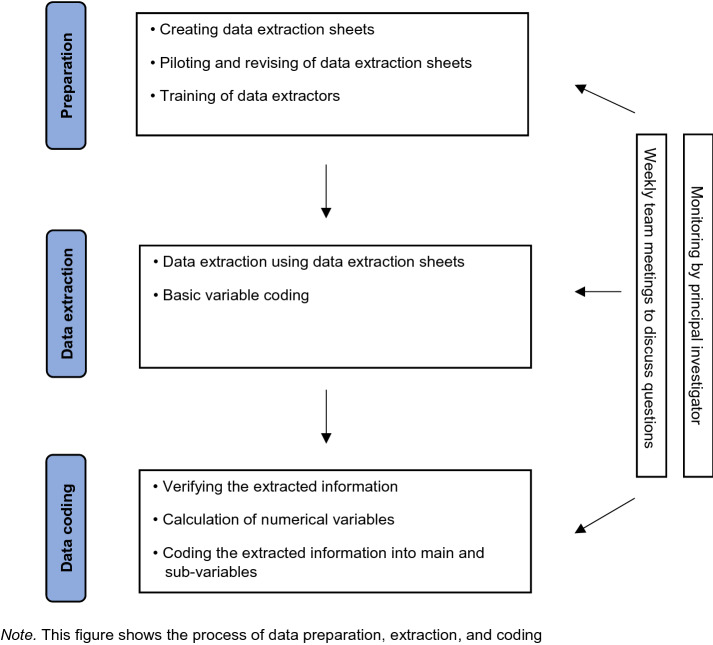
Data extraction and coding process of the retrospective cohort study

The retrospective chart review followed a standardized procedure of data extraction and coding (please see Fig. 2). Clinical parameters were retrieved from patients' paper files and computerized departmental documentation. Using a piloted data extraction template, four coders manually searched information from hospital admission, intra-treatment, and discharge records.

All patient data routinely collected and stored in the context of hospital treatment were anonymized for research purposes prior to evaluation. The data has been treated following the Data Protection Act 2017 and the General Data Protection Regulation [56, 58]. The Ethics Committee of the University Hospital Charité in Berlin, Germany, approved the retrospective chart review.

### Measures

Primary patient data were assessed, such as patient's sex, age at hospital admission, length of inpatient treatment, and psychiatric medication use during the hospital stay. In addition, information on EDs, NSSI, and psychiatric comorbidities was extracted.

*Eating disorders* The ED diagnosis was made upon hospital admission by a licensed child and adolescent psychiatrist or psychotherapist according to ICD-10 criteria [15]. In addition, the onset and duration of the ED, body weight in kilograms (kgs), and body height in meters (ms) at hospital admission and discharge were assessed from patient files. BMI percentiles at admission and discharge was computed as well as weight change in kgs per week [57, 58].

*Nonsuicidal self-injury* NSSI is conceived as the deliberate destruction of one's body skin without a conscious intent to die, with purposes socially not approved [4, 5, 59]. In this study, NSSI was based on multiple informants' reports upon hospital admission and during inpatient treatment.

Specifically, NSSI was systematically assessed as part of the patient's psychopathological history at hospital admission, based on the clinic's structured psychiatric history guide. Anamnestic information was gathered from the patient and parents/caregivers. In addition, a physical examination of the patient was conducted by a licensed physician, including the examination of skin damage and scars from NSSI.

Additionally, NSSI was documented as important biographical information if disclosed during treatment to any team member, including psychiatrists, psychotherapists, nurses, and other specialized staff. If NSSI was not documented in the patient file, we assumed its absence. For other coding decisions on missing data, please see Additional file 1: Table S1. For the retrospective review, information on NSSI from the patient files was encoded into two variables: lifetime prevalence and methods of NSSI.

*Psychiatric comorbidities* Psychiatric comorbidities were assessed upon hospital admission according to ICD-10 criteria [15]. Since patients were younger than 18 years, personality disorders were diagnosed as personality disorder *traits* if all criteria except age were met. The patients' intellectual abilities were rated according to documented clinical judgment in the following categories: Above average (IQ > 115), average (IQ 85–114), below average (IQ 70–84) [15].

At hospital admission, patient's history of suicidal ideation and suicide attempts was assessed as part of the medical history. Additionally, it was documented if a patient revealed current or past suicidal ideation or suicide attempts to any treatment team member during the hospital stay. Thinking about or planning suicide is defined as suicidal ideation [60]. A suicide attempt describes when one tries to end one's own life on purpose but survives [61]. Each behavior conforming to these criteria was extracted from the patients' records and coded as the variables suicidal ideation (yes/no) and suicide attempt (yes/no).

Additionally, the study coders gathered the following information from patient files: Presence of family psychopathology (yes/no), suicide or suicide attempt in the patient's environment (yes/no; environment: family, friends, acquaintance), history of experienced childhood abuse (yes/no; types: physical/sexual/emotional), and any type of psychiatric comorbidity.

### Statistical analysis

The initial sample comprised 415 patients (AN-R: n = 255 + AN-BP: n = 69 + AN subtype not specified: n = 7 + BN: n = 84). Altogether, 33 patients were excluded from the analyses due to the following reasons: AN subtype not specified (AN: n = 7), BMI percentiles in AN not < 10 at hospital admission (≠ ICD-10 criteria; AN-R: n = 6 + AN-BP: n = 11) or not available (AN-R: n = 2), regular menstruation in AN patients at hospital admission (≠ ICD-10 criteria; AN-R: n = 3), no data available to verify the inclusion criteria (AN-R: n = 2 + AN-BP: n = 2).

All statistical analyses were performed using the Statistical Package for Social Sciences (SPSS Version 27, [62]). The significance level was set at alpha = 0.05. The Kolmogorov–Smirnov test was used to test if the data were normally distributed. For non-normally-distributed variables, the Kruskal–Wallis-test was applied as the One Way Analysis of Variance (ANOVA's) non-parametric equivalent, and the Mann–Whitney U-test replaced t-tests. Of the 53 variables in the study dataset, 91% (n = 48) had no missing values across all ED subgroups. However, some missing values were identified in 9% (n = 5) of variables, with missing values ranging from 0.4 to 10.7%, except for body weight-related data in the BN group that were not the focus of treatment and that were missing in about half of the BN patients. We handled missing data by not omitting cases with missing data in one variable from all analyses but rather including them in those analyses for which data were available. No data were imputed.

First, we compared ED subgroups concerning sample characteristics and the lifetime prevalence of NSSI using ANOVAs for interval scaled variables and χ^2^-tests for nominal data. If the cell size was < 10, we applied a continuity correction; if the cell size was < 5, Fisher's exact test. We performed Tukey HSD and pairwise χ^2^-tests for post-hoc-testing. To explore the NSSI time trend, the following periods were grouped: 1990–1994, 1995–1999, 2000–2004, 2005–2009, 2010–2015. Further, within the ED subgroups, patients were categorized into and compared between those with vs. without lifetime NSSI. To test between-group differences, we computed independent-samples t-tests and χ^2^-tests to analyze the prevalence of NSSI and correlates. We calculated Cramer's phi for χ^2^-tests and Cohen's d for ANOVAs, t-tests, Man-Whitney-U, and Kruskal–Wallis tests to determine between-group effect sizes. All effect sizes were interpreted according to Cohen's [63] classification: no effect = 0.0–0.1, small effect = 0.2–0.4, intermediate effect = 0.5–0.7, large effect = 0.8- ≥ 1.0.

Finally, to identify independent correlates of NSSI, a stepwise forward binary logistic regression analysis was conducted for AN-R, AN-BP, and BN separately. NSSI (yes/no) was defined as the dependent variable for each ED subgroup. All variables that differed significantly at baseline and intra-treatment between the ED groups with versus without NSSI were included as potential mediators. We aimed to avoid multicollinearity to reach high reliability of the effects of the independent variables in the regression models. Therefore, we removed all but one variable if variables were highly correlated, if several variables provided similar results, or if one variable was computed from other data set variables. Nagelkerke's r squared metric was calculated to indicate the percent variance explained by the independent variables retained in the final model (explanatory quality: < 0.1 = small, 0.1–0.3 = intermediate, > 0.5 = large, [64]).

## Results

### Baseline sample characteristics

Altogether, 382 inpatients (females = 97.1%) were diagnosed with an ED, having a median age of 15.6 years (interquartile range: 14.3, 16.7; age range: 9–18 years). Table 1 presents the sample characteristics comparing AN-R (n = 242, 63.4%), AN-BP (n = 56, 14.7%), and BN (n = 84, 22.0%). ED subgroups differed significantly regarding patient age at ED onset and admission (youngest in AN-R), duration of illness (shortest in AN-R, longest in BN), and all anthropometric measures at admission (lowest in AN-R) (all p-values < 0.001).

**Table 1:** Sample characteristics and lifetime prevalence of nonsuicidal self-injury in patients with eating disorders

	Total (*N* = 382)	AN-R (*n* = 242)	AN-BP (*n* = 56)	BN (*n* = 84)	ANOVA Kruskal–Wallis-test χ^2^-test				Tukey HSD Mann–Whitney-U-test Pairwise χ^2^-tests		
	M ± SD Mdn (Q1,Q3) n (%)	M ± SD Mdn (Q1,Q3) n (%)	M ± SD Mdn (Q1,Q3) n (%)	M ± SD Mdn (Q1,Q3) n (%)	df_1_ df_2_	F H χ^2^	p	d phi	AN-R vs. AN-BP	AN-BP vs. BN	BN vs. AN-R
Sex	–	–	–	–	2	1.7	0.426	0.07	–	–	–
Female	371 (97.1)	233 (96.3)	55 (98.2)	83 (98.8)	–	–	–	–	–	–	–
Male	11 (2.9)	9 (3.7)	1 (1.8)	1 (1.2)	–	–	–	–	–	–	–
Age of ED onset, years	14.1 (13.2,15.3)	14.0 (12.9,15.0)	14.7 (13.6,15.9)	14.6 (13.5,15.7)	2	17.0	** < 0.001**	0.41	**0.001**	0.501	**0.002**
At hospital admission											
Duration of illness, months	13.9 ± 12.5	11.6 ± 11.0	16.6 ± 15.1	19.1 ± 13.0	2,367	12.7	** < 0.001**	**0.53**	**0.015**	0.471	** < 0.001**
Age, years	15.6 (14.3,16.7)	14.9 (13.8,16.3)	16.4 (15.3,17.1)	16.4 (15.5,17.2)	2	58.2	** < 0.001**	**0.83**	** < 0.001**	0.608	** < 0.001**
BMI percentiles	1.0 (1.0,5.0)	1.0 (1.0,1.0)	1.0 (1.0,2.0)	43.5 (19.5,73.5)	2	239.6	** < 0.001**	**2.67**	0.062	** < 0.001**	** < 0.001**
At hospital discharge											
BMI percentiles	11.0 (6.0, 18.0)	10.0 (5.0,16.5)	10.0 (4.0,16.0)	38.8 (17.0,74.3)	2	53.8	** < 0.001**	**0.86**	0.817	** < 0.001**	** < 0.001**
Weight change, kg/week	0.5 (0.3,0.7)	0.5 (0.4,0.7)	0.5 (0.3,0.6)	− 0.0 (− 0.3,0.2)	2	80.4	** < 0.001**	**1.09**	0.064	** < 0.001**	** < 0.001**
Treatment duration, days	92.0 ± 50.9	96.6 ± 42.5	100.5 ± 50.2	72.8 ± 67.3	2,376	7.9	** < 0.001**	0.41	0.853	**0.004**	**0.001**
Intelligence											
Above average	139 (36.4)	97 (40.1)	19 (33.9)	23 (27.4)	2	4.5	0.104	0.11	–	–	–
Average	235 (61.5)	142 (58.7)	36 (64.3)	57 (67.9)	2	2.4	0.296	0.08	–	–	–
Below average	8 (2.1)	3 (1.2)	1 (1.8)	4 (4.8)	2	3.8	0.149	0.10	–	–	–
Family psychopathology and history of childhood abuse											
Family psychopathology present	241 (63.1)	155 (64.0)	41 (73.2)	45 (53.6)	2	5.8	0.054	0.12	–	–	–
Suicide (attempt) environment	38 (9.9)	24 (9.9)	7 (12.5)	7 (8.3)	2	0.7	0.722	0.04	–	–	–
History of childhood abuse	44 (11.5)	15 (6.2)	11 (19.6)	18 (21.4)	2	18.4	** < 0.001**	0.22	**0.001**	0.798	** < 0.001**
Emotional	18 (4.7)	5 (2.1)	4 (7.1)	9 (10.7)	2	11.3	**0.004**	0.17	0.068	0.563	**0.002**
Physical	15 (3.9)	7 (2.9)	5 (8.9)	3 (3.6)	2	4.4	0.109	0.11	–	–	–
Sexual	13 (3.4)	3 (1.2)	4 (7.1)	6 (7.1)	2	9.4	**0.009**	0.2	**0.025**	1.000	**0.011**
Psychiatric comorbidities at hospital admission											
Average number	0.0 (0.0,1.0)	0.0 (0.0,1.0)	1.0 (0.0,1.0)	1.0 (0.0,1.0)	2	23.0	** < 0.001**	**0.49**	**0.001**	0.855	** < 0.001**
At least one	185 (48.4)	96 (39.7)	34 (60.7)	55 (65.5)	2	20.6	** < 0.001**	0.23	**0.004**	0.566	** < 0.001**
Substance abuse	7 (1.8)	0 (0.0)	3 (5.4)	4 (4.8)	2	13.4	**0.002**	0.18	**0.006**	1.000	**0.004**
Affective disorders, at least one	117 (30.6)	61 (25.2)	22 (39.3)	34 (40.5)	2	9.2	**0.010**	0.16	**0.034**	0.888	**0.008**
Persistent affective disorder	62 (16.2)	26 (10.7)	13 (23.2)	23 (27.4)	2	15.0	**0.001**	0.20	**0.013**	0.581	** < 0.001**
Major depression	61 (16.0)	37 (15.3)	12 (21.4)	12 (14.3)	2	1.5	0.471	0.06	–	–	–
Recurrent depressive episode	2 (0.5)	0 (0.0)	1 (1.8)	1 (1.2)	2	3.7	0.157	0.10	–	–	–
Anxiety disorders, at least one	11 (2.9)	8 (3.3)	2 (3.6)	1 (1.2)	2	1.1	0.574	0.05	–	–	–
Phobia	8 (2.1)	6 (2.5)	1 (1.8)	1 (1.2)	2	0.5	0.765	0.04	–	–	–
Panic disorder	4 (1.0)	2 (0.8)	1 (1.8)	1 (1.2)	2	0.4	0.808	0.03	–	–	–
Mixed anxiety and depression	2 (0.5)	1 (0.4)	0 (0.0)	1 (1.2)	2	1.1	0.586	0.05	–	–	–
Generalized anxiety disorder	1 (0.3)	1 (0.4)	0 (0.0)	0 (0.0)	2	0.6	0.748	0.04	–	–	–
Obsessive–compulsive disorder	30 (7.9)	23 (9.5)	5 (8.9)	2 (2.4)	2	4.5	0.107	0.11	–	–	–
Adjustment disorder	7 (1.8)	5 (2.1)	1 (1.8)	1 (1.2)	2	0.3	0.875	0.03	–	–	–
Post-traumatic stress disorder	2 (0.5)	1 (0.4)	1 (1.8)	0 (0.0)	2	2.2	0.331	0.08	–	–	–
Personality disorder traits	48 (12.6)	13 (5.4)	10 (17.9)	25 (29.8)	2	35.4	** < 0.001**	0.31	**0.004**	0.163	** < 0.001**
Borderline	27 (7.1)	3 (1.2)	7 (12.5)	17 (20.2)	2	37.2	** < 0.001**	0.31	** < 0.001**	0.336	** < 0.001**
Combined	7 (1.8)	2 (0.8)	1 (1.8)	4 (4.8)	2	5.3	0.068	0.12	–	–	–
Histrionic	5 (1.3)	3 (1.2)	0 (0.0)	2 (2.4)	2	1.5	0.473	0.06	–	–	–
Anankastic	4 (1.0)	4 (1.7)	0 (0.0)	0 (0.0)	2	2.3	0.311	0.08	–	–	–
Anxious	3 (0.8)	1 (0.4)	1 (1.8)	1 (1.2)	2	1.3	0.515	0.06	–	–	–
Mixed and other	2 (0.5)	0 (0.0)	1 (1.8)	1 (1.2)	2	3.7	0.157	0.10	–	–	–
Disorders with onset in childhood/adolescence	7 (1.8)	4 (1.7)	1 (1.8)	2 (2.4)	2	0.2	0.912	0.02	–	–	–
Lifetime history of nonsuicidal self-injury, suicidal ideation, and suicide attempts											
NSSI types, average number	0.0 (0.0,0.0)	0.0 (0.0,0.0)	0.0 (0.0,1.0)	0.0 (0.0,1.0)	2	10.5	**0.005**	**0.97**	** < 0.001**	0.409	** < 0.001**
At least one type of NSSI	82 (21.5)	20 (8.3)	22 (39.3)	40 (47.6)	2	69.6	** < 0.001**	0.43	** < 0.001**	0.527	** < 0.001**
Cutting	71 (18.6)	14 (5.8)	21 (37.5)	36 (42.9)	2	72.1	** < 0.001**	0.44	** < 0.001**	0.527	** < 0.001**
Scratching	10 (2.6)	4 (1.7)	1 (1.8)	5 (6.0)	2	4.7	0.095	–	–	–	–
Hitting	7 (1.8)	3 (1.2)	1 (1.8)	3 (3.6)	2	1.9	0.390	–	–	–	–
Burning	4 (1.0)	1 (0.4)	2 (3.6)	1 (1.2)	2	4.4	0.111	–	–	–	–
Suicidal ideation	117 (30.6)	48 (19.8)	25 (44.6)	44 (52.4)	2	37.2	** < 0.001**	0.31	** < 0.001**	0.370	** < 0.001**
Suicide attempt	13 (3.4)	4 (1.7)	5 (8.9)	4 (4.8)	2	7.9	**0.019**	0.14	**0.014**	0.484	0.211
Psychiatric medication prescription during treatment											
Average number	0.0 (0.0,1.0)	0.0 (0.0,0.0)	0.0 (0.0,1.0)	0 (0.0,0.8)	2	73.6	**0.001**	0.30	**0.001**	**0.015**	0.759
At least one	101 (26.4)	56 (23.1)	24 (42.9)	21 (25.0)	2	9.2	**0.010**	0.16	**0.003**	**0.027**	0.730
Antidepressants	76 (19.9)	36 (14.9)	21 (37.5)	19 (22.6)	2	15.1	**0.001**	0.20	** < 0.001**	0.056	0.103
Antipsychotics	51 (13.4)	32 (13.2)	12 (21.4)	7 (8.3)	2	5.0	0.082	0.11	–	–	–
Anxiolytics	2 (0.5)	2 (0.8)	0 (0.0)	0 (0.0)	2	1.2	0.559	0.06	–	–	–

AN-BP: anorexia nervosa, binge-purge type, AN-R: AN, restricting type, BN: bulimia nervosa; disorders with onset in childhood/adolescence comprise asperger syndrome, combined disorder of conduct and emotions, conduct disorders, elective mutism emotional disorders of childhood, reactive attachment disorder, stutter, tics; intelligence: above average: IQ > 115, average: IQ 85–114, below average: IQ 70–84; zero cases scored within the intellectual disability range (IQ < 70); Mdn: median, NSSI: nonsuicidal self-injury, SD: standard deviation, significant p-values ≤ 0.05 and effect sizes ≥ 0.45 (≥ medium effect size) are bold

Further, ED subgroups varied significantly in their prevalence of a history of childhood abuse, both regarding any history of childhood abuse (p < 0.001; BN:21.4% ≈ AN-BP:19.6% >> AN-R:6.2%), as well as involving childhood emotional abuse (p = 0.004; AN-BP:7.1% ≈ BN:10.7% > AN-R:2.1%), and childhood sexual abuse (p = 0.009; BN:7.1% ≈ AN-BP:7.1% >> AN-R:1.2%).

Moreover, the same pattern (BN ≈ AN-BP >> AN-R) was found regarding the following psychiatric variables: ≥ 1 psychiatric comorbidity (p < 0.001; BN:65.5% ≈ AN-BP:60.7% >> AN-R:39.7%), substance abuse (p = 0.002; BN:4.8% ≈ AN-BP:5.4% >> AN-R:0.0%), affective disorders (p = 0.010; BN:40.5% ≈ AN-BP:39.3% >> AN-R:25.2%), persistent affective disorder (p = 0.001; BN:27.4% ≈ AN-BP:23.2% >> AN-R:10.7%), and personality disorder traits (p < 0.001; BN:29.8% ≈ AN-BP:17.9% >> AN-R:5.4%), including borderline personality disorder traits (p < 0.001; BN:20.2% ≈ AN-BP:12.5% >> AN-R:1.2%).

### Inpatient treatment sample characteristics

ED subgroups differed significantly regarding inpatient treatment duration (longest in AN-R and AN-BP, shortest in BN) and the prevalence of taking ≥ 1 psychiatric medication (p = 0.010; AN-BP:42.9% >> BN:25.0% ≈ AN-R:23.1%), including antidepressants (p = 0.001; AN-BP:37.5% > BN:26.2% ≈ AN-R:14.9%). Moreover, ED subgroups differed significantly regarding all anthropometric measures at discharge (AN-R < / = AN-BP << BN) (all p-values < 0.001).

### Lifetime prevalence of nonsuicidal self-injury in anorexia nervosa and bulimia nervosa

Overall, 21.5% of patients with ED reported a history of NSSI, with 86.6% cutting, 12.2% scratching, 8.5% hitting, and 4.9% burning themselves. Lifetime NSSI was highest in BN (47.6%), intermediate in AN-BP (39.3%), and lowest in AN-R (8.3%) (p < 0.001). Specifically, NSSI was significantly lower in AN-R compared to AN-BP (p < 0.001) and BN (p < 0.001), while AN-BP and BN did not differ significantly (p = 0.527). The same pattern was observed regarding the average number of different NSSI types (p = 0.005), including cutting (p < 0.001; BN:42.9% ≈ AN-BP:37.5% >> AN-R:5.8%), scratching (p = 0.095), hitting (p = 0.390), and burning (p = 0.111). Over 25 years of the review, NSSI prevalence increased from 13% in the early 90 s to 28% in the 2010s across ED subgroups (please see Additional file 2: Fig. S1).

### Clinical correlates of nonsuicidal self-injury in anorexia nervosa and bulimia nervosa

Table 2 displays clinical correlations of NSSI in patients with AN-R, AN-BP, and BN, while Table 3 reports the respective effect sizes. In AN-R, NSSI was associated with a higher prevalence of psychiatric comorbidities, including a higher prevalence of ≥ 1 psychiatric comorbidity (NSSI + :85.0% vs. NSSI-:35.6%, p < 0.001), affective disorders (NSSI + :65.0% vs. NSSI-:21.6%, p < 0.001), major depression (NSSI + :35.0% vs. NSSI-:13.5%, p = 0.026), persistent affective disorder (NSSI + :40.0% vs. NSSI-:8.1%, p < 0.001), anxiety disorders (NSSI + :15.0% vs. NSSI-:2.3%, p = 0.021), personality disorder traits (NSSI + :20.0% vs. NSSI-:4.1%, p = 0.015), including histrionic personality disorder traits (NSSI + :10.0% vs. NSSI-:0.5%, p = 0.019).

**Table 2:** Clinical correlates of lifetime nonsuicidal self-injury in patients with eating disorders

	AN-R (*n* = 242)					AN-BP (*n* = 56)						BN (*n* = 84)				
	NSSI (*n* = 20)	No NSSI (*n* = 222)	t-test U-test χ^2^-test			NSSI (*n* = 22)	No NSSI (*n* = 34)		t-test U-test χ^2^-test			NSSI (*n* = 40)	No NSSI (*n* = 44)	t-test U-test χ^2^-test		
	M ± SD Mdn (Q1,Q3) n (%)	M ± SD Mdn (Q1,Q3) n (%)	df z	t U χ^2^	p	M ± SD Mdn (Q1,Q3) n (%)	M ± SD Mdn (Q1,Q3) n (%)		df z	t U χ^2^	p	M ± SD Mdn (Q1,Q3) n (%)	M ± SD Mdn (Q1,Q3) n (%)	df z	t U χ^2^	p
Sex	–	–	1	0.1	1.000	–	–		1	0.0	1.000	–	–	1	0.0	1.000
Female	20 (100)	213 (95.9)	–	–	–	22 (100)	33 (97.1)		–	–	–	40 (100)	43 (97.7)	–	–	–
Male	0 (0.0)	9 (4.1)	–	–	–	0 (0.0)	1 (2.9)		–	–	–	0 (0.0)	1 (2.3)	–	–	–
Age of ED onset, years	13.6 (12.7,14.8)	14.0 (12.9,15.0)	− 0.7	1886.5	0.463	14.1 (13.2,15.2)	15.1 (14.0,16.1)		− 1.8	269.5	0.080	14.2 (13.1,16.0)	14.8 (13.9,15,5)	− 1.2	593.0	0.244
At hospital admission																
Duration of illness, months	12.7 ± 10.6	11.5 ± 11.1	237	− 0.5	0.637	17.9 ± 14.9	15.8 ± 15.5		54	− 0.5	0.609	21.6 ± 14.0	16.5 ± 11.4	73	− 1.7	0.089
Age, years	15.1 (13.6,16.3)	14.9 (13.8,16.3)	− 0.1	2195.5	0.935	16.1 (15.2,17.0)	16.6 (15.5,17.2)		− 1.1	306.5	0.257	16.5 (15.3,17.2)	16.4 (15.6,17.2)	− 0.2	862.5	0.875
BMI percentiles	1.0 (1.0,1.0)	1.0 (1.0,1.0)	− 0.1	2198.0	0.910	1.0 (1.0,2.0)	1.0 (1.0,1.5)		− 0.4	352.5	0.643	50.0 (19.9,75.0)	36.0 (19.5,72.5)	− 0.3	694.0	0.775
At hospital discharge																
BMI percentile	7.5 (2.3,16.0)	10.0 (6.0,16.5)	− 1.6	1716.5	0.103	9.0 (5.5,15.8)	10.0 (3.0,17.0)		− 0.4	341.0	0.705	48.0 (17.5,75.5)	31.0 (14.0,72.0)	− 0.8	157.5	0.406
Weight change, kg/week	0.4 (0.3,0.4)	0.6 (0.4,0.7)	− 3.4	1184.5	**0.001**	0.4 (0.2,0.6)	0.5 (0.3,0.7)		− 1.5	274.0	0.126	− 0.0 (− 0.3,0.2)	0.0 (− 0.2,0.3)	− 0.6	265.0	0.558
Treatment duration, days	119.3 ± 35.7	94.5 ± 42.5	239	− 2.5	**0.012**	108.7 ± 50.9	95.2 ± 49.8		54	− 1.0	0.331	93.6 ± 86.1	52.9 ± 32.6	49.5	− 2.8	**0.007**
Intelligence																
Above average	9 (45.0)	88 (39.6)	1	0.2	0.641	8 (36.4)	11 (32.4)		1	0.1	0.757	15 (37.5)	8 (18.2)	1	4.0	0.055
Average	11 (55.0)	131 (59.0)	1	0.0	0.911	13 (59.1)	23 (67.6)		1	0.1	0.714	22 (55.9)	35 (79.5)	1	4.7	0.030
Below average	0 (0.0)	3 (1.4)	1	0.0	1.000	1 (4.5)	0 (0.0)		1	0.0	0.393	3 (7.5)	1 (2.3)	1	0.4	0.343
Family psychopathology and history of childhood abuse																
Family psychopathology present	17 (85.0)	138 (62.2)	1	3.2	0.073	15 (68.2)	26 (76.5)		1	0.1	0.708	23 (57.5)	22 (50.0)	1	0.5	0.491
Suicide (attempt) environment	3 (15.0)	21 (9.5)	1	0.2	0.430	1 (4.5)	6 (17.6)		1	1.1	0.226	4 (10.0)	3 (6.8)	1	0.0	0.704
History of childhood abuse	3 (15.0)	12 (5.4)	1	1.5	0.115	6 (27.3)	5 (14.7)		1	0.7	0.417	10 (25.0)	8 (18.2)	1	0.2	0.621
Physical	1 (5.0)	6 (2.7)	1	0.0	0.458	3 (13.6)	2 (5.9)		1	0.3	0.371	1 (2.5)	2 (4.5)	1	0.0	1.000
Emotional	1 (5.0)	4 (1.8)	1	0.0	0.353	1 (4.5)	3 (8.8)		1	0.0	1.000	5 (12.5)	4 (9.1)	1	0.0	0.730
Sexual	0 (0.0)	3 (1.4)	1	0.0	1.000	4 (18.2)	0 (0.0)		1	4.2	**0.020**	4 (10.0)	2 (4.5)	1	0.3	0.418
Psychiatric comorbidities at hospital admission																
Average number	1.0 (1.0,2.0)	0.0 (0.0,1.0)	− 4.8	980.0	** < 0.001**	1.0 (1.0,2.0)	0.0 (0.0,1.0)		− 2.6	228.0	**0.009**	1.0 (1.0,2.0)	0.5 (0.0,1.0)	− 3.6	507.0	** < 0.001**
At least one	17 (85.0)	79 (35.6)	1	16.7	** < 0.001**	18 (81.8)	16 (47.1)		1	5.4	**0.012**	33 (82.5)	22 (50.0)	1	9.8	**0.002**
Substance abuse	0 (0.0)	0 (0.0)	–	–	–	2 (9.1)	1 (2.9)		1	0.2	0.555	1 (2.5)	3 (6.8)	1	0.2	0.618
Affective disorders, at least one	13 (65.0)	48 (21.6)	1	16.1	** < 0.001**	11 (50.0)	11 (32.4)		1	1.7	0.187	21 (52.5)	13 (29.5)	1	4.6	**0.032**
Persistent affective disorder	8 (40.0)	18 (8.1)	1	16.3	** < 0.001**	7 (31.8)	6 (17.6)		1	0.8	0.367	13 (32.5)	10 (22.7)	1	1.0	0.316
Major depression	7 (35.0)	30 (13.5)	1	5.0	**0.026**	5 (22.7)	7 (20.6)		1	0.0	1.000	8 (20.0)	4 (9.1)	1	1.2	0.215
Recurrent depressive episode	0 (0.0)	0 (0.0)	–	–	–	1 (4.5)	0 (0.0)		1	0.0	0.393	1 (2.5)	0 (0.0)	1	0.0	0.476
Anxiety disorders, at least one	3 (15.0)	5 (2.3)	1	5.8	**0.021**	1 (4.5)	1 (2.9)		1	0.0	1.000	1 (2.5)	0 (0.0)	1	0.0	0.476
Phobia	2 (10.0)	4 (1.8)	1	2.3	0.080	0 (0.0)	1 (2.9)		1	0.0	1.000	1 (2.5)	0 (0.0)	1	0.0	0.476
Panic disorder	1 (5.0)	1 (0.5)	1	0.8	0.159	1 (4.5)	0 (0.0)		1	0.0	0.393	1 (2.5)	0 (0.0)	1	0.0	0.476
Mixed anxiety and depression	1 (5.0)	0 (0.0)	1	2.3	0.083	0 (0.0)	0 (0.0)		–	–	–	1 (2.5)	0 (0.0)	1	0.0	0.476
Generalized anxiety disorder	0 (0.0)	1 (0.5)	1	0.0	1.000	0 (0.0)	0 (0.0)		–	–	–	0 (0.0)	0 (0.0)	–	–	–
Obsessive–compulsive disorder	2 (10.0)	21 (9.5)	1	0.0	1.000	2 (9.1)	3 (8.8)		1	0.0	1.000	2 (5.0)	0 (0.0)	1	0.6	0.224
Adjustment disorder	0 (0.0)	5 (2.3)	1	0.0	1.000 1 (4.5)		0 (0.0)		1	0.0	0.393	0 (0.0)	1 (2.3)	1	0.0	1.000
Post-traumatic stress disorder	0 (0.0)	1 (0.5)	1	0.0	1.000	1 (4.5)	0 (0.0)		1	0.0	0.393	0 (0.0)	0 (0.0)	–	–	–
Personality disorder traits	4 (20.0)	9 (4.1)	1	6.3	**0.015**	7 (31.8)	3 (8.8)		1	3.4	**0.038**	19 (47.5)	6 (13.6)	1	9.9	**0.002**
Borderline	1 (5.0)	2 (0.9)	1	0.3	0.229	4 (18.2)	3 (8.8)		1	0.4	0.415	12 (30.0)	5 (11.4)	1	3.4	0.064
Combined	1 (5.0)	1 (0.5)	1	0.7	0.159	1 (4.5)	0 (0.0)		1	0.0	0.393	4 (10.0)	0 (0.0)	1	2.7	**0.047**
Histrionic	2 (10.0)	1 (0.5)	1	7.0	**0.019**	0 (0.0)	0 (0.0)		–	–	–	2 (5.0)	0 (0.0)	1	0.6	0.224
Compulsive	0 (0.0)	4 (1.8)	1	0.0	1.000	0 (0.0)	0 (0.0)		–	–	–	0 (0.0)	0 (0.0)	–	–	–
Anankastic	0 (0.0)	1 (0.5)	1	0.0	1.000	1 (4.5)	0 (0.0)		1	0.0	0.393	1 (2.5)	0 (0.0)	1	0.0	0.476
Mixed and other	0 (0.0)	0 (0.0)	–	–	–	1 (4.5)	0 (0.0)		1	0.0	0.393	0 (0.0)	1 (2.3)	1	0.0	1.000
Disorders with onset in childhood/adolescence	0 (0.0)	4 (1.8)	1	0.0	1.000	1 (4.5)	0 (0.0)		1	0.0	0.393	0 (0.0)	2 (4.5)	1	0.4	0.495
Suicidal ideation	12 (60.0)	36 (16.2)	1	19.5	** < 0.001**	15 (68.2)	10 (29.4)		1	6.6	**0.010**	27 (67.5)	17 (38.6)	1	7.0	**0.008**
Suicide attempt	1 (5.0)	3 (1.4)	1	1.5	0.239	5 (22.7)	0 (0.0)		1	5.9	**0.007**	3 (7.5)	1 (2.3)	1	0.4	0.343
Psychiatric medication prescription during treatment																
Average number	0.0 (0.0,1.0)	0.0 (0.0,0.0)	− 2.5	1670.5	**0.013**	1.0 (0.0,2.0)	0.0 (0.0,1.0)		− 2.5	239.0	**0.011**	0.0 (0.0,1.0)	0.0 (0.0,0.0)	− 3.5	586.0	**0.001**
At least one	9 (45.0)	47 (21.2)	1	4.6	**0.032**	14 (63.6)	10 (29.4)		1	5.1	**0.024**	17 (42.5)	4 (9.1)	1	10.8	**0.001**
Antidepressants	7 (35.0)	29 (13.1)	1	5.3	**0.021**	12 (54.5)	9 (26.5)		1	3.4	0.066	15 (37.5)	4 (9.1)	1	8.1	**0.003**
Antipsychotics	5 (25.0)	27 (12.2)	1	1.6	0.201	8 (36.4)	4 (11.8)		1	3.5	**0.045**	6 (15.0)	1 (2.3)	1	2.9	**0.050**
Anxiolytics	1 (5.0)	1 (0.5)	1	0.7	0.159	0 (0.0)	0	(0.0)	–	–	–	0 (0.0)	0 (0.0)	–	–	–

AN-BP: anorexia nervosa, binge-purge type, AN-R: AN, restricting type, BN: bulimia nervosa; disorders with onset in childhood/adolescence comprise asperger syndrome, combined disorder of conduct and emotions, conduct disorders, elective mutism emotional disorders of childhood, reactive attachment disorder, stutter, tics; intelligence: above average: IQ > 115, average: IQ 85–114, below average: IQ 70–84; Mdn: median, NSSI: nonsuicidal self-injury, SD: standard deviation, significant p-values ≤ 0.05 are bold

**Table 3:** Effect sizes of correlates of lifetime nonsuicidal self-injury in patients with eating disorders

	AN-R d phi	AN-BP d phi	BN d phi
Sex	0.06	0.11	0.11
Age of ED onset, years	0.10	**0.48**	0.27
At hospital admission			
Duration of illness, months	0.11	0.14	0.40
Age, years	0.01	0.31	0.03
BMI percentile	0.01	0.10	0.07
At hospital discharge			
BMI percentile	0.22	0.10	0.27
Weight change, kg/week	**0.45**	0.42	0.17
Treatment duration, days	**0.59**	0.27	**0.63**
Intelligence			
Above average	0.03	0.04	0.21
Average	0.02	0.09	0.26
Below average	0.03	0.17	0.12
Family psychopathology and history of childhood abuse			
Family psychopathology present	0.13	0.09	0.08
Suicide (attempt) environment	0.05	0.19	0.06
History of childhood abuse	0.11	0.15	0.08
Physical	0.04	0.13	0.06
Emotional	0.06	0.08	0.06
Sexual	0.03	0.35	0.11
Psychiatric comorbidities at hospital admission			
Average number	**0.55**	**0.69**	**0.78**
At least one	0.28	0.35	0.34
Substance abuse	–	0.01	0.10
Affective disorders, at least one	0.28	0.18	0.23
Persistent affective disorder	0.28	0.16	0.11
Major depression	0.16	0.03	0.16
Recurrent depressive episode	–	0.17	0.12
Anxiety disorders, at least one	0.20	0.04	0.12
Phobia	0.15	0.11	0.12
Panic disorder	0.14	0.17	0.12
Mixed anxiety and depression	0.22	–	0.12
Generalized anxiety disorder	0.02	–	–
Obsessive–compulsive disorder	0.01	0.01	0.16
Adjustment disorder	0.04	0.17	0.11
Disorders with onset in childhood/adolescence	0.04	0.17	0.15
Post-traumatic stress disorder	0.02	0.17	-
Personality disorder traits	0.20	0.29	0.37
Borderline	0.10	0.14	0.23
Combined	0.14	0.17	0.24
Histrionic	0.23	–	0.16
Compulsive	0.04	–	–
Anxious	0.02	0.17	0.12
Mixed and other	–	0.17	0.11
Suicidal ideation	0.30	0.38	0.29
Suicide attempt	0.08	0.39	0.12
Psychiatric medication prescription during treatment			
Average number	0.23	**0.64**	**0.60**
At least one	0.16	0.34	0.39
Antidepressants	0.17	0.28	0.34
Antipsychotics	0.10	0.29	0.23
Anxiolytics	0.14	–	–

AN-BP: anorexia nervosa, binge-purge type, AN-R: AN, restricting type, BN: bulimia nervosa, bolded effect sizes ≥ 0.45 (≥ medium effect size)

Furthermore, AN-R patients with NSSI (AN-R-NSSI +) reported a higher prevalence of suicidal ideation (NSSI + :60% vs. NSSI-:16.2%, p < 0.001). Also, AN-R-NSSI + status was associated with a significantly lower weekly weight gain (kg: p = 0.001), longer treatment duration (NSSI + :119.3 ± 35.7 days vs. NSSI-:94.5 ± 42.5 days, p = 0.012), and a greater likelihood of receiving ≥ 1 psychiatric medication (NSSI + :45.0% vs. NSSI-:21.2%, p = 0.032), namely antidepressants (NSSI + :35% vs. NSSI-:13.1%, p = 0.021).

Similarly, the AN-BP-NSSI + group evidenced more patients suffering from ≥ 1 psychiatric comorbidity at hospital admission (NSSI + :81.8% vs. NSSI-:47.1%, p = 0.012), particularly personality disorder traits (NSSI + :31.8% vs. NSSI-:8.8%, p = 0.038). In addition, AN-BP-NSSI + was associated with a significantly higher prevalence of a history of childhood sexual abuse (NSSI + :18.2% vs. NSSI-:0.0%, p = 0.020), suicidal ideation (NSSI + :68.2% vs. NSSI-:29.4%, p = 0.010), and suicide attempts (NSSI + :22.7% vs. NSSI-:0.0%, p = 0.007). AN-BP-NSSI + status was associated with a greater likelihood of receiving ≥ 1 psychiatric medication (NSSI + :63.6% vs. NSSI-:29.4%, p = 0.024), namely antipsychotics (NSSI + :36.4% vs. NSSI-:11.8%, p = 0.045).

Similar trends were also observed in BN patients, with NSSI status being associated with a significantly greater prevalence of having ≥ 1 psychiatric comorbidity at hospital admission (NSSI + :82.5% vs. NSSI-:50.0%, p = 0.002), including affective disorders (NSSI + :52.5% vs. NSSI-:29.5%, p = 0.032), personality disorder traits (NSSI + :47.5% vs. NSSI-:13.6%, p = 0.002), namely combined personality disorder traits (NSSI + :10.0% vs. NSSI-:0.0%, p = 0.047), and suicidal ideation (NSSI + :67.5% vs. NSSI-:38.6%, p = 0.008). BN-NSSI + status was also associated with a significantly longer inpatient treatment duration (NSSI + :93.6 ± 86.1 days vs. NSSI-:52.9 ± 32.6 days, p = 0.007) and a greater likelihood of receiving ≥ 1 psychiatric medication (NSSI + :42.5% vs. NSSI-:9.1%, p = 0.001), namely antidepressants (NSSI + :37.5% vs. NSSI-:9.1%, p = 0.003), and antipsychotics (NSSI + :15.0% vs. NSSI-:2.3%, p = 0.050).

### Independent correlates of nonsuicidal self-injury

Independent correlates of NSSI in AN-R were less weekly weight gain (OR = 0.03, 95%CI = 0.02–0.43, p = 0.001), a higher prevalence of psychiatric comorbidities (OR = 2.93, 95%CI = 1.42–6.04, p = 0.004), and suicidal ideation (OR = 0.21, 95%CI = 0.72–0.64, p = 0.006) (Nagelkerke's r^2^ = 0.39). In AN-BP, a higher prevalence of psychiatric comorbidities was independently associated with NSSI (OR = 2.67, 95%CI = 1.13–6.31, p = 0.025) (Nagelkerke's r^2^ = 0.45). In BN, NSSI independently correlated with a higher prevalence of psychiatric comorbidities (OR = 3.75, 95%CI = 1.71–8.23, p = 0.001) and a longer inpatient treatment duration (OR = 1.01, 95%CI = 1.00–1.02, p = 0.033) (Nagelkerke's r^2^ = 0.33) (Table 4).

**Table 4:** Logistic regression analyses for baseline and intra-treatment variables of nonsuicidal self-injury in eating disorders

ED	Nagelkerke r^2^, overall model	Variables	OR (95% CI)	B (SE)	Wald	df	p
AN-R	0.39	Weight change kg/week	0.03 [0.02, 0.43]	− 3.67 (1.44)	6.52	1	**0.001**
(n = 242)		Psychiatric comorbidities, average number	2.93 [1.42, 6.04]	1.08 (0.37)	8.46	1	**0.004**
		Suicidal ideation (0 = no, 1 = yes)	0.21 [0.72, 0.64]	− 1.54 (0.56)	7.68	1	**0.006**
AN-BP (n = 56)	0.45	Psychiatric comorbidities, average number	2.67 [1.13, 6.31]	0.99 (0.44)	5.00	1	**0.025**
BN	0.33	Psychiatric comorbidities, average number	3.75 [1.71, 8.23]	1.32 (0.40)	10.87	1	**0.001**
(n = 82)		Treatment duration	1.01 [1.00,1.02]	0.01 (0.00)	4.54	1	**0.033**

Significant p-values ≤  0.05 are bold

AN-BP: anorexia nervosa, binge-purge type, AN-R: AN, restricting type, BN: bulimia nervosa; B: beta, CI: confidence interval, ED: eating disorder, OR: odds ratio, SE: standard error

## Discussion

Youth inpatients suffering from EDs who also engage in NSSI represent a particularly vulnerable group with increased suicide risk. However, basic epidemiological data are lacking. To close this gap, we analyzed lifetime NSSI prevalence and clinical correlates in a large sample of 382 youth inpatients, split by ED subtypes, AN-R, AN-BP, and BN. In this retrospective chart review, across ED subgroups, more than one out of five individuals engaged in NSSI. Patients with a lifetime history of NSSI were characterized by a higher prevalence of psychiatric comorbidities, psychotropic medication use, and suicidal ideation.

### Lifetime prevalence of nonsuicidal self-injury in anorexia nervosa and bulimia nervosa

The lifetime NSSI prevalence of 22% in the sample we studied is comparable to the meta-analytically derived lifetime NSSI prevalence of 27% in adolescents and adults with EDs managed in various treatment settings [20]. As hypothesized, NSSI was most prevalent in BN, followed by AN-BP and AN-R. High levels of impulsivity and negative affect are more common in binge-purge than restricting EDs. At the same time, impulsivity and negative affect also underlie NSSI, which may explain a higher prevalence among AN-BP and BN patients [38, 39].

Concerning AN-R, we found an NSSI prevalence of 8%, which is lower than the previously reported 32% lifetime NSSI estimate in an adolescent and young adult outpatient sample with AN-R [32]. Peebles et al.'s (2011) retrospective cohort study is the only analysis we know, focusing on lifetime NSSI in adolescents and young adults with AN-R, yet these were outpatients. The scarcity of prior research in this population makes prevalence comparisons difficult. Therefore, it remains unclear if the prevalence in our study underestimates the true prevalence. Potential reasons for such a hypothetical underestimation could be a lower NSSI disclosure in youth AN-R inpatients. For AN-BP, we observed lifetime NSSI in 40%, well in line with the prevalence of 43% reported by outpatients [32]. Compared with two other studies on lifetime NSSI prevalence in BN outpatients, our sample's lifetime NSSI prevalence of 48% in BN was approximately twice as high ([30]: 29%, [32]: 21%). One likely explanation for the elevated prevalence estimates in our BN subsample is that patients had a comparatively high disease severity, as reflected by an increased prevalence of comorbidities and prolonged treatment duration.

Taken together, compared with studies of adolescent and adult ED outpatients, the NSSI lifetime prevalence estimates in our study were lower in AN-R, consistent in AN-BP, and higher in BN. However, for all we know, no prior research investigated lifetime NSSI in youth inpatients divided by ED subgroups AN-R, AN-BP, and BN, which would be required for a proper comparison. To better understand lifetime NSSI in hospitalized ED youths, more epidemiological studies in this specific group are needed.

Concerning NSSI methods, the youth inpatients with AN-R, AN-BP, and BN that we analyzed were all primarily engaged in cutting (87%), scratching, and hitting themselves (9–12%). This ranking of NSSI methods is in line with a review including individuals of different ages in the community and psychiatric in- and outpatients [40], as well with previous research from Paul et al. [41], who investigated an adult AN and BN inpatient sample (mean age = 24.3 ± 7.1 years). Hence, the NSSI method engaged in appears to be similar across various age groups, diagnoses, and settings.

Over time, in our sample of youth ED inpatients, lifetime NSSI prevalence doubled from the 1990s to the 2010s. This time trend aligns with clinical experience and is expected to increase further during today's COVID-19 pandemic. Specifically, under COVID-19, the prevalence of NSSI among adolescents appears to have increased compared to the 2010s [14]. Studies on the current course of NSSI prevalence in young inpatients with EDs are needed.

### Clinical correlates of nonsuicidal self-injury in anorexia and bulimia nervosa

Across ED subgroups of inpatients, a high prevalence of psychiatric comorbidities accompanied NSSI, which is in line with previous findings [11, 13, 31]. Specifically, in AN-R and BN, NSSI was correlated with major depression and personality disorder traits, and in AN-R, additionally with anxiety disorder, in agreement with findings in community and adult ED populations [3, 13, 31, 37, 44–46]. Moreover, in AN-BP, lifetime NSSI prevalence was associated with a history of childhood sexual abuse. These correlations align with previous findings [3, 43–45] and can be well explained by the emotion-regulation function of NSSI: Engaging in NSSI is described as reducing negative emotions and tension that are common in depression, anxiety, and personality disorders [2, 19]. Further, childhood maltreatment is hypothesized to create vulnerability for emotion dysregulation [19].

In AN-R and BN, NSSI was associated with a longer inpatient treatment duration and, in AN, also with a lower weekly weight gain. Together these findings indicate that NSSI is linked to higher disease severity. In addition, NSSI was associated with a higher prevalence of psychotropic medication prescription, particularly antidepressants in AN-R, antipsychotics in AN-BP, and both medication groups in BN. This observation aligns with previous research linking NSSI with increased prevalence of antidepressant and antipsychotic medication prescription [47, 48], clinical severity, and medical help-seeking [21].

Alarmingly, across ED subgroups, NSSI was associated with a higher prevalence of suicidal ideation and suicide attempts in AN-BP patients, in line with previous research [2, 3, 10, 11, 19, 45]. While NSSI and suicidality are on a spectrum of self-destructive behaviors [3, 19], NSSI is assumed to facilitate committing suicide due to repeated pain exposure and the related reversal from primary experienced fear to relief [24, 25].

ED-specific multivariable regression analyses revealed independent correlations of lifetime NSSI, adjusted for other significant NSSI associates in the final statistical model. The final model revealed a higher prevalence of psychiatric comorbidities for all ED subgroups as an independent correlate of NSSI. Additionally, a higher prevalence of suicidal ideation and less weekly weight gain in AN-R, and longer treatment duration in BN were independently associated with NSSI. These findings again underscore that more severely ill patients engage in NSSI. Altogether, the clinical correlates of NSSI in this study are consistent with findings from the community [10, 11, 43], as well as psychiatric [2, 19] and ED-specific populations [3, 13, 37, 44–46]. As far as we know, this study is the first to provide NSSI correlates specifically for youth inpatients and the ED subgroups, AN-R, AN-BP, and BN, which is necessary to tailor prevention and treatment efforts aiming at more effective care.

### Limitations and future research

This study benefits from a relatively large sample of consecutively admitted patients with different ED diagnoses. The sample included a broad age range from 9 to 18 years. In addition, our analyses comprised detailed information on ED subtypes, psychiatric comorbidities, psychotropic medication use, and anthropometric characteristics for hospital admission and discharge.

However, our study is not without limitations: First, data were acquired by chart review, providing only routinely assessed clinical parameters. Standardized diagnostic assessments or questionnaires were not applied. Therefore, psychological parameters, such as the underlying intentions and effects of engaging in NSSI, were not accessible. These factors would promote a better understanding of why this subpopulation engaged in NSSI and how to best address this dysfunctional behavior. Second, although we included information on the specific type of NSSI, data on the age of onset, frequency, and severity of NSSI were not available. Further, since we assumed NSSI as absent, if it was not documented in the patient file, it is possible that our coding led to an underestimation of its prevalence. To better understand the phenomenology of NSSI, future research should examine such details using standardized psychological questionnaires and interviews. Third, this study explored the time course of NSSI prevalence only descriptively and across ED subtypes. Detailed inference statistics comparing ED subtypes would require a sufficiently large sample, which is beyond the scope of this retrospective review. Fourth, this study only evaluated data until the year 2015. More up-to-date data on NSSI in psychiatric ED inpatients are needed, as the NSSI prevalence in adolescents appeared to increase over time, especially since the COVID-19 pandemic started. Fifth, even if treatment plans for EDs and NSSI remained similar across time, variability in the implementation of care, depending on the therapist and over time, cannot be ruled out. Sixth, this study focused on the patients' first inpatient stay and did not analyze subsequent hospital readmissions since the sample size of readmission was small, and readmission may be linked to covariates, such as disease chronicity. Seventh, causal relationships between EDs, psychiatric comorbidities, and NSSI cannot be inferred due to the retrospective cross-sectional design. Eighth, our sample was not representative of all ED patients but more likely reflective of a more severe and complex subgroup of patients with EDs. Therefore, the generalizability of the study results is limited to other patient subgroups. For example, we deliberately focused on inpatients with typical EDs. Hence it is unclear to what degree the study findings can be generalized to youth with EDs who do not require hospitalization or suffer from atypical EDs. To address in sufficient detail NSSI in the heterogeneous subgroups of non-typical EDs, we are preparing a separate manuscript on NSSI in atypical AN, atypical BN, and EDNOS [65]. Further, the patients studied were treated at a highly specialized University Hospital in a large city. Therefore, the study findings may not be generalized to youth ED populations in non-specialized psychiatric inpatient treatment. A large multicenter study, including ED populations receiving non-specialized versus specialized psychiatric inpatient care in rural versus urban areas, would be desirable to compare various ED populations and treatment settings. Ninth, the data quality may be limited by some degree of missing values, introducing a risk of selection bias, decreasing statistical power, and potentially reducing the validity of the reported findings. In this context, socioeconomic data could not be explored as 47–68% of patients had missing data across the relevant variables. Finally, data quality is limited if not all coders generated the identical coding of patient records, which remains unclear since we were not able to obtain measures of interrater reliability.

However, despite these limitations, to our knowledge, this is the first study reporting on the relationship between specific ED subtypes and NSSI in child and adolescent inpatients, which also focused on the type of NSSI and independent correlates. More research on the precursors and predictors of NSSI is required to improve prevention programs and treat modifiable risk factors adequately. Overall, longitudinal epidemiological studies of NSSI in the vulnerable population of inpatients with AN-R, AN-BP, and BN and NSSI are needed, including sex- and age-group-specific analyses, preferably in a multicenter study setting.

## Conclusions

Across ED subgroups, we found a high lifetime prevalence of NSSI accompanied by an increased prevalence of psychiatric comorbidities and suicidal ideation. These findings indicate the need for standardized routine screening of NSSI in youth with EDs, especially in AN-BP and BN. Moreover, early prevention programs and tailored interventions by specialized staff are needed for this population. In addition to focusing on the ED as the primary diagnosis, assessing and treating comorbidities and NSSI is crucial. Further, suicidality should be considered in youth inpatients with EDs and NSSI to improve patient care and suicide prevention.

## Supplementary Information

**Additional file 1: Figure S1. ** 25-year time course of the prevalence of nonsuicidal self-injury in patients with eating disorders.

**Additional file 2: Table S1.** Coding decisions to deal with missing data per variable.

## Data Availability

The dataset generated and analyzed during the current study is not publicly available because of privacy and ethical restrictions due to the high data protection of children and adolescents' data in psychiatric institutions in Germany. However, they are available from the corresponding author on reasonable request.

## Abbreviations

AN-BP: Anorexia nervosa, binge-purge type
ANOVA: Analysis of variance
AN-R: Anorexia nervosa, restricting type
BN: Bulimia nervosa
EDs: Eating disorders
ICD-10: International Classification of Diseases, 10th edition
NSSI: Nonsuicidal self-injury
