# Supplementary material for: Lifetime prevalence and clinical correlates of nonsuicidal self-injury in youth inpatients with eating disorders: a retrospective chart review
Source: Child Adolesc Psychiatry Ment Health. 2022 Feb 28;16:17. doi: 10.1186/s13034-022-00446-1 (PMC8884089; doi:10.1186/s13034-022-00446-1)
Supplement: Supplementary file 1 — Additional file 1: Figure S1. 25-year time course of the prevalence of nonsuicidal self-injury in patients with eating disorders. [file 13034_2022_446_MOESM1_ESM.docx]

**Supplementary Table 1** Coding decisions to deal with missing data per variable

| **Variable** |  | **Coding decisions made to deal with missing data** |
| --- | --- | --- |
| Sex |  | If no information on the sex of the patient was available in the patient file, we coded "999=missing data". |
|  |  |  |
| Age of ED onset |  | If no information on the age of the ED onset was available in the patient file, we coded "999=missing data". |
|  |  |  |
| Duration of illness at hospital admission |  | If no information on the duration of illness was available in the patient file, we coded "999=missing data". |
|  |  |  |
| Age, years |  | If no information on the age of the patient at hospital admission was available in the patient file, we coded "999=missing data". |
|  |  |  |
| BMI percentiles at hospital admission or discharge |  | If no information on the bodyweight of the patient was available at hospital admission or discharge in the patient file, we coded "999=missing data". |
|  |  |  |
| Intelligence |  | If no information on the intelligence of the patient was available in the patient file, we coded "999=missing data". |
|  |  |  |
| Family psychopathology present |  | If neither the psychiatrist, the psychotherapist, nor any other treatment team member documented family psychopathology, we assumed no family psychopathology was present. Accordingly, we coded "0=no family psychopathology present". |
|  |  |  |
| Suicide (attempt) environment |  | If neither the psychiatrist, the psychotherapist, nor any other treatment team member documented an attempted or committed suicide in the environment of the patient, we assumed no suicide (attempt) was present. Accordingly, we coded "0=no suicide (attempt) in the environment". |
|  |  |  |
| History of childhood abuse |  | If neither the psychiatrist, the psychotherapist, nor any other treatment team member documented a history of childhood abuse, we assumed no history of childhood abuse. Accordingly, we coded "0=no history of childhood abuse". |
|  |  |  |
| Psychiatric comorbidities |  | We assumed no psychiatric comorbidities if the psychiatrist and the psychotherapist documented no psychiatric comorbidities. Accordingly, we coded "0=no psychiatric comorbidities". |
|  |  |  |
| Nonsuicidal self-injury |  | If neither the psychiatrist, the psychotherapist, nor any other treatment team member documented nonsuicidal self-injury, we assumed no nonsuicidal self-injury. Accordingly, we coded "0=no nonsuicidal self-injury". |
|  |  |  |
| Suicidal ideation |  | If neither the psychiatrist, the psychotherapist, nor any other treatment team member documented suicidal ideation, we assumed no suicidal ideation. Accordingly, we coded "0=no suicidal ideation". |
|  |  |  |
| Suicide attempt |  | If neither the psychiatrist, the psychotherapist, nor any other treatment team member documented a suicide attempt, we assumed no suicide attempt. Accordingly, we coded "0=no suicide attempt". |
|  |  |  |
| Psychiatric medication prescription |  | If no psychiatric medication prescription was mentioned in the patient file, we assumed no psychiatric medication was prescribed and coded "0=no psychiatric medication". |

*Note.* This table shows coding decisions made to deal with missing data for each variable
