# Supplementary material for: Lifetime prevalence and clinical correlates of nonsuicidal self-injury in youth inpatients with eating disorders: a retrospective chart review
Source: Child Adolesc Psychiatry Ment Health. 2022 Feb 28;16:17. doi: 10.1186/s13034-022-00446-1 (PMC8884089; doi:10.1186/s13034-022-00446-1)
Supplement: Supplementary file 2 — Additional file 2: Table S1. Coding decisions to deal with missing data per variable. [file 13034_2022_446_MOESM2_ESM.docx]

**Supplementary Figure 1** 25-year time course of the prevalence of nonsuicidal self-injury in patients with eating disorders

*Notes.* NSSI=history of nonsuicidal self-injury; the variables *patients with NSSI* and *period of hospital admission* refer to patients ≤18 years admitted to the child and adolescent inpatient unit of the University Hospital Charité in Berlin, Germany, with an ICD-10 diagnosis of anorexia nervosa, restrictive type, anorexia nervosa, binge-purge type, and bulimia nervosa.
